# Collectanea

**Published:** 1843-09

**Authors:** 


### Collectanea.

### CURE OF ABSCESS OF THE TOOTH.

*To the Editor of the Lancet*:—Sir.—In the Lancet of Dec. 3, 1842, page 99, you published an article upon "intra-dental abscess," with remarks on the "use of a vent in the stopping of decayed teeth." As I am of opinion that you would wish to correct error on the subject, I have thought that a communication from this side of the Atlantic would not prove unacceptable. Your valuable publication being now republished here by one of our most enterprising firms, that of Messrs. Wilson and Co. many persons in America have the advantage of perusing its columns, and thus the above article came under my notice, when perceiving at once the palpable mistake that it contains, I determined to explain the unsoundness of the theory, and the uselessness of putting in practice the propositions therein named.

Mr. Saunders (the author of the paper) says, that when the internal cavity of a tooth is suddenly exposed (the nerve and its membrane, of course, is to be understood) by the breaking down of the thin layer of carious bone which protected it, by "injudiciously" stopping it, inflammation and suppuration speedily supervene. He afterwards proposes, in order to obviate the difficulty of intra-dental abscess, to plug up the cavity in the tooth, and drill a hole through the "stopping" so as to reach into the cavitas pulpæ, and thus form "a little canal" as an outlet for the pus secreted therein, as well as for the tooth's pent-up nervous irritability. The theory is ingenious, but the practice unfortunately does not preserve the tooth, nor will protect its owner from tooth-ache.

Mr. Ghrimes, in the Lancet of Dec. 17, 1842, satisfactorily proves the mechanical uselessness of the "little canal," but unfortunately offers, as an amendment to the method proposed by Mr. S. a proposition which is worse than useless, namely, that of drilling a hole from the neck of the tooth, through its substance, into the internal cavity, to answer as a substitute for the little "canal of Saunders." This also has the same objection, that it will not prevent or arrest the decaying of the tooth, nor prevent its aching, while, in addition, it would allow the decay to undermine the stopping, so that perchance the patient would one day discover that with his meal he had swallowed the crown of his tooth, leaving the "stump" in its place. It is dangerous to the tooth to meddle with its neck in any way. Ample proof of this fact is offered in the serious injury done to sound teeth, where clasps, wires, or ligatures are applied around their necks to secure artificial teeth, the baneful practice of some dentists. The neck of the tooth, externally, is its most tender and vital part.

Mr. Saunders candidly admits that his practice does not effect a cure; nor did he attempt it, believing it to be, as he says, "impossible, at least in the present state of dental surgery," to arrest the suppurative process when once established "under such circumstances and in such a situation." With all due deference to Mr. Saunders' experience, and his opinions of "the present state of dental surgery," I beg to state that not only an arrest, but also a perfect cure, may and can be effected, and the tooth effectually stopped, and rendered useful for life. The following formula.

R Ox. arsen. alb, 120th gr.;

Pulv. gall. ¼ gr.;

Opii, gr. ss; made into a paste, and applied into the cavity of the tooth, and kept there either by a softened piece of wax, or a pledget of lint saturated with kreosote, will entirely remove it, in one application. All that is necessary afterwards, for two or three days, is to apply a little dry sulphate of lime, introduced into the tooth to absorb the little corruption left there. The tooth may then be sponged out with eau de Cologne, and filled or stopped, either with gold foil or a paste composed of marmora and silica. I have followed this practice for fourteen years, and with the exception of two cases within that time I have never failed to eradicate the disease, and effect a perfect and permanent cure. I remain, sir, your most obedient servant,

New York, Feb. 25, 1843. A. C. Castle, M. D. *Dentist.*

[London Lancet.

### DENTAL ABSCESS.—FASTENING.

*To the Editor:—Sir,*—I feel indebted to Mr. Castle, of New York, for his account of the treatment that he recommends for the cure of abscess in the tooth. That gentleman, however, should have remembered that I did not offer my method as a *cure*, but, at the best, only as a palliative; and the circumstance of my stating a case which had been successful for about two years, seems entirely to have escaped his notice. The discussion of this subject, however, having been the means of bringing forward a *cure*, I would solicit your attention to another point. Mr. Castle, as an objection to my method, remarks, that the danger of meddling with the neck of a tooth, in any way, is amply shown by "the serious injury done to sound teeth, where clasps, wires, or ligatures are applied around their necks, to secure artificial teeth, the baneful practice of some dentists." Although the mechanical part of the dental profession has been brought to a very high state of perfection, still, sir, I have yet to learn a plan by which artificial teeth can be supplied without recourse to the means of attaching them to others, when there are any remaining, (I allude to the *generality* of cases.) That such can be avoided is commonly asserted by advertisers, but these very men, in most cases, having but little knowledge of their profession, eventually use them, to the detriment of the other teeth. If the clasp be properly adjusted to the adjoining teeth, and its pressure be equally applied upon that part of the surface which it covers, it will *not* be injurious. This assertion of Mr. Castle not being attributable to the same motive as our London *professors*, and Mr. C. having shown his wish to disseminate information, he would, I am sure, confer a favour upon the profession generally, if he favoured us with an explanation of his system of accomplishing this desideratum in London practice. I am, sir, your obedient servant,

SAMUEL GHRIMES, *Dentist.*

Baker st. March 29, 1843. [London Lancet.

*Staphyloraphy.*—The *Dublin Journal of Medical Science,* for January last, contains an account of three cases, in which the operation for uniting the cloven soft palate was performed successfully. The operator in the first case was Dr. Cusack, surgeon to Stevens' Hospital, in the two others Sir P. Crampton. Dr. Cusack's patient was a medical student, eighteen years of age, labouring under a congenital fissure of the soft palate. For some time previous to the operation he was directed to employ measures to diminish the irritability of the fauces, the existence of which constitutes one of the principal difficulties, and in the removal of which he was perfectly successful.

Dr. Cusack proceeded as follows:—With the aid of a simple forceps and curved needles, three ligatures were passed at equal distances from each other through the soft palate, the lowest being at the base of the uvula; a double-edged knife was then introduced about a line from the margin of the cleft, and the same distance from the apex of the triangle; on each side in succession, the incisions being terminated above. After the lapse of a short time, during which the patient took some light nourishment, the edges of the wound were approximated, and the ligatures tied with a surgical knot; one of them, however, having been cut, as it was suppose, too closely, unravelled, and was replaced. Some slight hæmorrhage followed, with teazing cough, and a few hours after the operation all the ligatures had become unravelled, and were, of necessity, replaced by two others at points more remote from the margins of the fissure. They were each secured by a simple knot. No untoward symptoms occurred afterwards, and on the fifth day the ligatures were cut, the palate being perfect, the only remaining defect being a bifid uvula, a condition commonly met with in persons who articulate with perfect distinctness.

Sir Philip Crampton's patients were a boy of twelve years of age and a young lady aged sixteen. The peculiarity in the treatment of these cases consisted, first, in the manner of securing the ligature; and secondly, in the management of the patient after the operation. The difficulty of tying the second knot on the ligature, without suffering the first to become opened by the strong retraction of the edges of the fissure effected by the muscles of the palate, has always been acknowledged. This difficulty, however, was effectually removed by an ingenious contrivance of Mr. Maclean's; after the ligatures had been passed through the palate at the distance of a quarter of an inch from the cut edge of the fissure, and brought out at the mouth, their ends were passed through a small perforated metallic bead, such as are used in making purses; the bead was then pushed down along the ligatures, closing them as it descended, until it touched the approximated edges of the wound; it was then compressed with a pair of strong, blunt-pointed forceps, and the ligatures were thus firmly secured without a knot at the required degree of tension. The other and most important peculiarity in the treatment consisted in allowing the patients an ample supply of soft food during the whole period of the treatment. Boiled bread and milk, custard, soup, and jelly, were given twice or thrice a day, and the patients were not confined to their beds, Sir Philip Crampton conceiving that the total privation of all nourishment for five days, so strongly insisted on by Roux, was not only unnecessary, but in the highest degree unfavourable to the successful issue of the operation, as it must cause a state of constitutional disturbance highly unfavourable to the establishing of the healthy process of union by the first intention, in proof of which the observations of Messrs. Manoury and Thore, house-pupils at the Hotel-Dieu, where Roux's operations were performed, are quoted; they state that they have seen delirium and severe nervous derangement follow such protracted abstinence from food.—*Prov. Med. Jour.* March 25, 1843.—*Am. Jour. of Med. Sciences.*

*Elongated Uvula and Enlarged Tonsils.*—James Yearsley, Esq. a London surgeon, somewhat distinguished for his researches into the causes of deafness, has produced a treatise on *Elongated Uvula and Enlarged Tonsils,* which developes some important views. He points out the intimate connection between certain morbid conditions of the throat and ear, says the editor of the London Sun, and several imperfections of the voice and speech. Mr. Yearsley has established that when those parts are in a state of active inflammation, they may be excised with safety and even advantage, as there is scarcely any hæmorrhage. It is advisable, therefore, even in cases where it seems to be hazardous to perform the operation, not to hesitate a single moment, when the condition of the patient is at all endangered by the delay. Mr. Yearsley conceives that a relaxation of the mucous membrane of the throat and elongation of the uvula, give rise to symptoms which indicate a pulmonary consumption. An incessant irritation produced by an elongated, pendulous uvula, produces cough, and, if long continued, may develope a disease of the lungs, especially in persons hereditarily disposed to that malady. The voice of singers is singularly influenced by the uvula; if too long, the character of the voice is immediately improved by snipping it off. Practitioners should look to that little appendage often, since it may give rise to a multitude of formidable affections that might be removed instantly with a pair of scissors.—*Boston Med. and Surg. Jour.*

*Stammering.*—Dr. ABERCROMBIE read a paper on this subject to the Medico-Chirurgical Society of Edinburg in January last. When his attention was first directed to the subject, he stated, the following facts attracted his notice:—

I. He observed that stammerers never stammer in *singing*.

II. The individual on whom his first observations were made, did not stammer when he was obliged to speak in a louder tone of voice than usual, as when conversing in the midst of a noisy crowd, or in a carriage on a rough road.

III. The precentor of a church came under his notice who stammered in common conversation, but showed no hesitation when *reading out the line*, as it is called, which was done in a peculiar high-pitched tone of voice, such as is usually employed by precentors for that purpose.

IV. He found that stammerers have no difficulty in performing any of those movements of the lips and the tongue, by means of which the consonant sounds are produced, when they are directed to make these movements simply, that is, without any reference to speech

V. He observed, that in some stammerers, the difficulty is not confined to the consonant sounds, in which the peculiar action of the organs of speech is more directly exerted, but extends to other sounds, in which these organs are little, if at all concerned, such as the simple aspirated *h*, as in the words *happiness, holiness*, &c. In one individual, indeed, who was treated successfully, he found that he often hesitated at such words as these, long after he had overcome every difficulty respecting the consonants.

By such facts as these he was led to the conjecture, that the affection does not depend upon any defect in the organs of speech, properly so called; but is rather connected with a deficiency in the management of the voice; and he thought it would be found, that, when a stammerer gets into that peculiar state of hesitation, which is so familiar to every one who has witnessed it, he is endeavouring to speak when *he has no voice*; that is, when the lungs have become emptied of air, or nearly so.

According to these views, the principles on which the cure of the affection may be accomplished, appeared to be the following: In actually accomplishing a cure, every thing depends upon the perseverance of the patient himself after the principles have been explained to him.

I. To direct the attention of the individual to the three distinct parts, of which the function of speech consists, viz.

1. A full and continuous current of *air* proceeding outwards from the lungs.

2. The formation of this into *inarticulate sound, or voice*, by the action of the larynx.

3. The formation of this into *articulate sound*, or *speech*, chiefly by certain movements of the *lips* and the *tongue*.

He soon perceives that he has no difficulty in performing any of these actions, when they are thus made separate objects of attention; and in this manner he is led to understand that his affection does not depend upon any defect in any of the organs of speech, or a difficulty of performing any of the processes of which the function consists; but in a certain want of harmony among these processes, which has grown into a habit. He is easily made to perceive, for example, that he has no difficulty in performing that motion of the lips by which is formed the sound of the letter B, then why should he have any difficulty in saying *bee, boy, bell,* &c. When the formation of each letter is thus made a separate object of attention, or a distinct voluntary act, it is remarkable to observe how the difficulty seems to vanish; and by continued attention in this manner, the habit is gradually broken, in as far as concerns this part of the process.

II. The second, and principal part of the treatment is, to exercise the individual in the habit of never attempting to speak without having A FULL AND STRONG CURRENT OF VOICE. He may be made sensible of the effect of this, by making him read in a strong loud tone of voice, as if he were calling to a person at a distance,—or in a tone resembling singing or chanting,—or in the peculiar tone of a precentor, in reading out the line, which has been already referred to. When he has thus been made to understand the principles on which the removal of the affection is to be conducted, the farther treatment consists in a course of exercises calculated to give him a full command of his voice, and so to correct the habit which he has acquired, of speaking, or attempting to speak, without sufficient voice. For this purpose he should be made to read aloud, several times a-day, from an author whose style is somewhat declamatory. In doing so, he should be made to read in a high-pitched tone, and to stop frequently and take a full breath, so as to have the voice thrown out with a force beyond what is required for ordinary reading or ordinary conversation. With this view it is necessary to make him stop and take a full inspiration much more frequently than would be required by another person; for it is in this part of the process that we are to trace, in a great measure, the bad habit which he has acquired, and the opposite habit which he is required to cultivate. In particular, whenever he feels the tendency to hesitate at a word, he is to be taught to stop instantly, take a full breath, and then try it again. He will be immediately sensible of the effect; and a succession of voluntary efforts of this kind will be gradually formed into a habit, calculated to correct the injurious habit, in which, I believe, we are chiefly to trace the pathology of stammering.

In a note appended to this paper, Dr. Abercrombie remarked, that since his observations were written, he had found that the same principle, respecting the influence of respiration in this affection, had been pointed out by Dr. M'Cormack of Belfast, in a small volume published in 1828.—*London and Ed. M. J. Med. Sci.* March, 1843.—*Am. Jour. of Med. Sciences.*

*Science of Meteorology.*—A highly complimentary notice of Dr. Forry's late work on meteorology, by the celebrated philosopher Humboldt, has been published. It appears he regretted that he could not employ for his comparisons of Asiatic climatology with the American, Dr. Forry's judicious researches on the climate of the United States and its endemic influences. A variety of approbatory remarks have also appeared from elevated sources in this country, which must be gratifying to an author, since it is so often the case that a prophet has but little reputation at home.—*Boston Med. and Surg. Jour.*

---

*Regeneration of the Teeth after Caries of the Upper Jaw-Bone.*—A boy, eleven years old, was, after the suppression of tinea, affected with a painful swelling of the upper jaw-bone of the right side; the teeth became loose, and numerous abscesses formed, through which a probe could be passed into the antrum. The right nasal cavity was compressed by the swelling of the bone, and the eye forcibly pushed upwards. The canine and first molar teeth being extracted, and an abscess at the internal angle of the eye opened, there was an abundant purulent discharge, which was followed by the exfoliation of the os unguis, and of part of the processus nasalis maxill. superior; the abscesses in the gums discharged also osseous fragments. In this manner seventy-two pieces of bone were exfoliated; their total weight was 126 grains, and they consisted of the alveolar process; the anterior and external paries, and the nasal process of the upper jaw bone; the os unguis, and the nasal bone of the right side. After four months, the ulcerations began to heal; the patient's general health improved; the swelling of the face subsided, and the eye regained its natural position; in this state he remained for eight months, when he was again attacked with pain in the posterior part of the alveolar process; and with swelling of the gums; after an incision in the latter, the pain diminished; no pus was found; but within a few days, three molar teeth were protruded; and two months afterwards another appeared. Since that time the patient has enjoyed very good health; no more teeth have been formed, but the new ones have remained in good condition.—*Graefe u. Walther's Jour.*—*British Quar. Jour. of Dent. Surg.*

---

*Artesian Wells.*—We understand that it is intended to carry the bore for the Artesian Well in the Garden of Plants to the depth of 800 or 900 metres, whereas that at Grenelle is only 550 metres deep. The object of piercing so low is to find water of a high temperature. The expectation of doing so is founded on observations made by M. Arago and M. Walferdin at Grenelle, that the temperature of the water increased in warmth one degree at every 32 metres depth, and consequently at that of 800 or 900 metres must be at from 36 to 39 degrees centigrade, (about from 97 to 104 Fahrenheit,) with which the hot-houses of the equatorial plants, and also the lodges of the animals in the menagerie, and even the hospitals in that quarter, may be warmed in winter.—*French paper.*—*N. York Jour. of Med.*

*Dental Caries.*—This affection, so lamentable on account of the violent pain which it occasions, and its ordinary result the premature loss of the teeth, has numerous causes. But the recent labours of several pathologists authorize the conclusion, that in most cases caries may be ascribed to the presence of the acid liquid secreted by the glands of the gums, about the neck of the teeth. Experiments conducted with care, have shown that all the other secretions from the mouth, have a more or less decided alkaline character. This liquid is distinguished from them by a constant acidity which seems to acquire more activity in those chronic affections of the stomach, manifested by Anorexia, and weakness of the digestive organs. Nothing is more common, in fact, than the loss of teeth, after those long indispositions which can hardly be named diseases. It is rare, however, that the inferior incisors are altered, while the superior, on the contrary, are very subject to this change. Does not this remarkable fact point out that caries would be still more frequent were it not for the abundant secretion of saliva of an alkaline character. The inferior incisores, constantly bathed by the saliva, do not experience the effects produced by the acid secretion from the gums, to the same degree as the others, as the saliva by its presence neutralizes the acidity of this liquid, The useful conclusion to be drawn from this fact is, that in the daily attention bestowed upon the teeth, nothing is more clearly indicated than the use of dentifrices, in which the alkaline principle predominates.—*Archives de la Medecine Belge.—British Quar. Jour. of Dent. Surg.*

*Suppression of Hœmorrhage at the Gum.*—By Thomas Embling, Esq.—The following case, illustrative of the great difficulty often experienced in stopping hæmorrhage from the minute maxillary arteries after the extraction of a tooth, will also show the efficacy of a simple method of cure in such cases, when pressure can be positively applied to the mouths of the bleeding vessels.

I was sent for, some time back, to a lady, who, on the preceding evening, had a tooth extracted by a dentist, and whose gum had continued to bleed profusely from the time of the removal of the tooth until I saw her, being a period of about eight hours.

I found the mouth filled by coagula, and a perpetual dripping of arterial blood escaping from the mouth. A variety of remedies had suggested themselves to the patient and her friends, but none had at all subdued the bleeding. On clearing the mouth thoroughly from the coagula, I observed that in the extraction of the tooth (the third molar of the upper jaw) the dentist had broken off a considerable portion of the alveolar process, leaving a point of bone sticking out in the hole which had been thus made. A large piece of the gum had been also torn away. Several minute arteries were bleeding freely in the gum. I employed all the usual remedies adopted in such cases, but none of them, neither lunar caustic, nitric acid, nor the actual cautery, effected a cessation of the hæmorrhage.

After using such remedies as I could devise for three or four hours, I tried the effect of compressing the part between the finger and the thumb. This produced great nausea and vomiting at first, but having no other agent upon which I could at all depend, I determined to try the effect of long-continued pressure upon the mouths of the bleeding vessels. Of course, during the immediate pressure of the finger and thumb, no hæmorrhage could occur, as the part allowed a direct application to the open vessels; but at the end of an hour there was very slight, if any, diminution of the hæmorrhage; in another hour, however, the decrease of hæmorrhage was decidedly perceptible; in a third hour still greater improvement was evident, and by five hours and a half after I first employed pressure the hæmorrhage had ceased entirely.

For some weeks the gum was extremely tender, but gradually the tenderness has passed off, although at the present time the spicula of bone which was left of the alveolar process (for I did not deem it prudent at all to increase the loss of bone) is very painful when pressed upon suddenly and violently.—*London Lancet.—Boston Med. and Surg. Jour.*

*Illegal Practice of Medicine in Paris.*—Some months ago a trial took place before the Tribunal of Correctional Police in Paris, which abundantly shows the jealousy with which the authorities view persons practising medicine in the French metropolis without having previously procured the necessary certificates of competency.

The case was that of a lady in Paris, who had been so severely bitten by a dog as to be upwards of a month confined to her bed, and in great suffering, in consequence of which she brought an action against the owner of the dog, and recovered 500 francs damages. The defendant, however, appealed against the verdict on the plea that the original injury had been greatly aggravated by the bad treatment of the medical man whom the lady called in, and proved by a physician's evidence that the remedies used were calculated to irritate instead of allaying the painful symptoms. By these means he succeeded in materially lowering the amount of damages claimed by the lady; and at the same time the Tribunal having decided that the medical man (who was a recognized *officier de santé* for the Dep. Saone et Loire) had acted illegally in practising his profession in Paris without special license for the same, issued an order for his prosecution.—*Lon. Lancet.*

*Potatoes a preventive and cure for Scurvy.*—Much has been said of late in France and England of the value of this vegetable, in the prevention and cure of scorbutic disease, administered several times a day, in its *raw state,* but scraped sufficiently fine to make it digestible. It seems to have been amply tested among the seamen of the French navy. In the United States army, however, this is no new remedy in scorbutus. Thus, in the first quarter of 1821, there were sixteen cases reported at Fort Crawford, Prairie du Chien, of which two terminated fatally. The medical officer, in his report, according to the "Medical Statistics of the United States Army," speaking of the employment of "*raw potatoes and vinegar,*" says:—"I selected four or five of the worst cases, which had received no alleviation from the use of the nitre and vinegar, and directed each one to eat per day a common soup-plate full of the potato sliced down in a sufficient quantity of vinegar. It had an immediate effect on the stomach, which recovered its natural vigor; the bowels became regular, the pains abated, the stricture of the tendons was overcome, the ulcers put on a healthy aspect, and, in a few days, the patient found himself in a happy state of convalescence."—*N. York Jour. of Med.*

*Dental Surgery in the United States.*—The subject of dental surgery appears to attract considerable attention among our friends on the western shores of the Atlantic. A school of dental surgery has lately been established, and is now in full activity, at Baltimore, superintended by four professors. Candidates, after an attendance on the lectures during two years, (or for a less time if they should have previously attended medical lectures,) undergo an examination and write a thesis on dental manipulations; besides which, they are required to perform certain operations, and to make artificial teeth, palates, &c. which, if approved of, they receive the degree of doctor of dental surgery. We heartily desire that some such anxiety for improving this branch of the profession in this country should be evinced.—*London Lancet.*

*An English General Practitioner.*—On a cottage window near Plymstock is the following:—"I ——— Parish Clark Seargeant, Smith, tacheth yong Garls and Buoys to rade and rite daleth in mole candals shugar plums rishlites comes, mole traps, mouse traps, spring guns, and all other sich matters—teeth distracted, blid drawn, blisters, Pils, mixturs maid, also nails, and hosses shoed, hepsome salts, and cornes cut, and all other things on rasonable Tarmes.—N. B. and also my Misses goes out has man whidwife in the cheepest way posuble."—*Dub. Med. Press.*—*N. York Jour. of Med.*

*Medical Things in Paris.*—It is rumored that Dr. F. Campbell Stewart, of New York, late family physician of Gen. Cass while a resident abroad, will soon publish a statistical account of the hospitals of Paris, together with memoirs of the most eminent French surgeons of the present day. No writer could have a more ample field to exercise in than this. If it is not an interesting and useful book, the author can make no satisfactory apology, so abundant and curious are the materials.—*Boston Med. and Surg. Jour.*

Mrs. M—, a lady, about twenty-two years of age, applied to me, in consequence of having suffered for eight months, severe pain in the branches of the superior and inferior maxillary nerves, coming at first in irregular paroxysms: latterly however they were periodical, and invariably came on at nine o'clock, A. M. and at seven, P. M. The severity of the attack generally lasted about an hour. It rarely occurred at any other time with violence, unless the patient was suffering from indisposition or mental agitation. At the early part of her suffering, she was induced by the recommendation and persuasion of a friend, to apply brandy and salt, mustard poultices, &c. but without effect. She consulted a physician of the highest character, who ordered the various preparations of iron in combination with quinine, which was continued for two months, but failed to give relief. Belladonna was now prescribed, commencing with one grain night and morning; one hour before the paroxysm came on, leeches and blisters were applied to the temple, with fomentations of poppies and camomile, with an increase of half-a-grain of belladonna at each dose, until three grains were taken twice a day. She now became so affected with sickness, vertigo, dimness of sight, &c. that this treatment was discontinued, and the ioduret of potass substituted, with the use of veratrine externally. These means were followed for six weeks without success. It was now suggested by a medical friend, that the disease possibly originated from diseased teeth. On examining the inferior maxilla on the left side, I perceived that the whole of the teeth from the canine to the dens sapientiæ were affected with caries, also the first and second molares in the superior maxilla, all of which were evidently producing considerable irritation in the surrounding parts. On these teeth being pressed, the paroxysms returned with the usual violence. I immediately removed the two bicuspides, and two molares of the lower jaw, and ordered Acet. Morphia, quarter of a grain; Mist. Camph, one ounce and a half; with the following aperient, Ext. Colocynth co. gr. vi. Hyd. Submur. gr. ii. in two pills,—with considerable relief. In the course of a week she was sufficiently recovered from the effect of the operation, to submit to have the other diseased teeth removed,—the dens sapientiæ of the lower, and the first and second molares of the upper jaw. The morphia draught and pills were continued for a few days, since which she has had no return of pain. A few months since I had the satisfaction of hearing that she was quite recovered, and had not experienced a moment's pain in the jaw, since the last operation.—*British Quar. Jour. of Dent. Surg.*

*Accident to Baron Berzelius.*—This very talented and eminent chemist has recently had a narrow escape with his life, from an explosion which occurred during some experimental investigation in which he was engaged. Although the injuries sustained by him are fortunately slight, yet, seeing the extent of mischief produced by the accident, it is a matter of surprise that he did not pay the forfeit of his life.—*N. York Jour. of Med.*

